# Fecal and Clinical Profiles of Dogs With Chronic Enteropathies Treated With Bile Acid Sequestrants for 5–47 Months: A Retrospective Case Series

**DOI:** 10.1111/jvim.70206

**Published:** 2025-08-20

**Authors:** Linda Toresson, Amanda B. Blake, Chi‐Hsuan Sung, Gunilla Olmedal, Ulrika Ludvigsson, Paula R. Giaretta, M. Katherine Tolbert, Jan S. Suchodolski

**Affiliations:** ^1^ Gastrointestinal Laboratory, Department of Small Animal Clinical Sciences, Texas A&M University College Station Texas USA; ^2^ Evidensia Specialist Animal Hospital Helsingborg Sweden

**Keywords:** bile acid diarrhea, bile acid dysmetabolism, cholestyramine, colesevelam, non‐responsive enteropathy

## Abstract

**Background:**

Bile acid (BA) malabsorption and BA diarrhea (BAD) are prevalent but underdiagnosed conditions in people with chronic diarrhea of multiple causes. Recent studies have shown BA dysmetabolism in dogs with chronic enteropathies (CE).

**Objective:**

Describe canine inflammatory bowel disease activity index (CIBDAI), dysbiosis index (DI) and fecal BA concentrations in healthy dogs and dogs with refractory or partially immunosuppressive‐responsive CE or CE dogs requiring high doses of corticosteroids, treated with BA sequestrants (BAS).

**Animals:**

Twenty‐four CE dogs and 18 healthy dogs.

**Methods:**

Retrospective case series. Dogs with CE were treated with BAS as adjunctive treatment. Fecal BA was analyzed using liquid chromatography–tandem mass spectrometry. Fecal microbiota was assessed using DI. Response to treatment was defined as a decrease in CIBDAI category.

**Results:**

Sixteen of 24 dogs improved clinically after BAS treatment (cholestyramine, 44–133 mg/kg q12h; colesevelam, 7–24 mg/kg q12h; colestipole, 26 mg/kg q24h). Duration of treatment was 5–47 months. Median (range) CIBDAI decreased from 6.5 (4–13) to 3 (2–5) in responders. At baseline, responders had a higher median (range) percentage of fecal unconjugated primary BAs (93 [1–100]) versus non‐responders (2.5 [0–95]) and healthy dogs (1.5 [0–19]). Dysbiosis index (median [range]) was higher in responders (4.2 [−4.7 to 9.0]) versus non‐responders (−0.1 [−1.5 to 5.7]) and healthy dogs (−4.5 [−6.4 to −0.2]).

**Conclusions and Clinical Importance:**

Treatment with BAS as adjunctive treatment potentially may benefit some dogs with nonresponsive or partially immunosuppressive‐responsive enteropathy or CE dogs requiring high doses of corticosteroids.

**Abbreviations**
ASBT: apical sodium‐dependent bile acid transporter
BA: bile acids
BAD: bile acid diarrhea
BAM: bile acid malabsorption
BAS: bile acid sequestrants
CE: chronic enteropathy
CE+BASR: chronic enteropathy responding to bile acid sequestrants
CE−BASR: chronic enteropathy non‐responsive to bile acid sequestrants
CI: confidence interval
CIBDAI: canine inflammatory bowel disease activity index
DI: dysbiosis index
ESAHHS: Evidensia Specialist Animal Hospital, Helsingborg, Sweden
f1BA: fecal primary bile acids
f2BA: fecal secondary bile acids
fBA: fecal bile acids
FMT: fecal microbiota transplantation
HSDH: hydroxysteroid dehydrogenase
IBS‐D: irritable bowel syndrome with diarrhea
IRE: immunosuppressive‐responsive enteropathy
NRE: non‐responsive enteropathy
P‐IRE: partially immunosuppressive‐responsive enteropathy
PLE: protein‐losing enteropathy
RI: reference interval

## 1 Introduction

Primary bile acids (1BA) are detergent molecules that are synthesized in the liver, conjugated to glycine or taurine, stored in the gallbladder, and released into the duodenum to facilitate fat digestion. Approximately 95% of 1BA are reabsorbed in the ileum by the apical sodium‐dependent BA transporter (ASBT) and undergo enterohepatic circulation [1]. Unabsorbed 1BA are deconjugated to cholic acid and chenodeoxycholic acid and dehydroxylated by 7α‐dehydroxylating colonic bacteria (mainly *Peptacetobacter* [previously *Clostridium*] *hiranonis* in dogs) to the secondary BAs (2BA), deoxycholic acid and lithocholic acid [2, 3, 4]. In healthy dogs and people, the majority of fecal BA are 2BA [5, 6, 7].

Decreased absorptive capacity of the ileum, accelerated transit time, and overproduction of BAs can result in BA malabsorption (BAM) and BA diarrhea (BAD) [8, 9, 10]. Furthermore, intestinal dysbiosis with a lack of 7α‐dehydroxylating colonic bacteria leads to decreased conversion to 2BAs and can generate BAD [7]. Despite a high prevalence of BAD in patients with Crohn's disease and diarrhea‐associated irritable bowel syndrome, BAD is an underrecognized and undertreated condition [11, 12], mainly because of a lack of diagnostic tools. Consequently, clinical response to empirically‐administered BA sequestrants (BAS) often is used to diagnose BAD in people.

Recent studies suggest that BA dysmetabolism occurs also in dogs. In a preliminary study, serum concentrations of 7α‐hydroxy‐4‐cholesten‐3‐one (C4), a metabolite of BA synthesis, were significantly increased in 3/17 dogs with refractory chronic diarrhea [13]. Supranormal serum C4 concentrations are indicative of BAM in people [14, 15]. Furthermore, BA dysmetabolism or decreased expression of ASBT in the ileum of dogs with chronic enteropathy (CE) has been reported [5, 6, 7, 16, 17]. A subset of dogs with CE had significantly lower amounts of fecal 2BA (f2BA) and increased percentage and amount of fecal 1BA (f1BA) compared to healthy dogs. The percentage of f2BA significantly increased over time in CE dogs responding to diet or corticosteroids [5, 17]. Intestinal dysbiosis has been reported in parallel with BA dysmetabolism in dogs with CE, typically characterized by increased dysbiosis index (DI) [18] and decreased abundance of the bile acid‐converting bacterium *P. hiranonis* [5, 6, 16]. Only one case report is available on treatment of suspected BAD in dogs [19]. Two dogs with chronic refractory diarrhea responded to cholestyramine, a BAS. The positive clinical effect in these dogs has led us to prescribe BAS to some dogs with CE when traditionally used treatment protocols have failed, or when high corticosteroid doses associated with marked adverse effects were required to control disease [20, 21]. The aim of our retrospective study was to describe clinical features and fecal variables in healthy dogs, non‐ or partially‐responsive CE dogs, and CE dogs requiring high doses of corticosteroids, responding to versus not responding to BAS.

## 2 Materials and Methods

### 2.1 Study Design and Ethics Approval

Ours was a retrospective study based on a review of medical records at the Evidensia Specialist Animal Hospital in Helsingborg, Sweden (ESAHHS) from November 2019 to March 2025. Naturally voided baseline fecal samples from CE dogs were stored at −20°C. In Sweden, formal ethical approval is not required for storing and using naturally‐ voided fecal samples from dogs for research purposes. However, 8/24 dogs were included in a prospective study on fecal microbiota transplantation (FMT). These dogs responded poorly to FMT and subsequently were started on BAS, according to the study protocol. The FMT study was approved by the animal ethics committee of Uppsala University, Uppsala, Sweden (5.8.18–138 777/2021; approved 24 September 2021).

A decrease of CIBDAI category after treatment (i.e., from severe or moderate to mild or insignificant and from mild to insignificant) was regarded as a response to BAS. This approach is similar to the one used in a study on corticosteroid‐refractory CE dogs treated with cyclosporine [22]. Canine inflammatory bowel disease index (CIBDAI) [23], fecal bile acids (fBA), DI, and abundance of *P. hiranonis* based on qPCR analysis [18] were compared between BAS responders and non‐responders. For comparison, data from 18 healthy staff‐owned dogs from ESAHHS were included after informed owner consent.

### 2.2 Study Population, Treatment and CIBDAI

Inclusion criteria were privately‐owned dogs with chronic diarrhea treated with BAS (cholestyramine, colesevelam, or colestipol) at the ESAHHS, with a baseline fecal sample collected before intervention. Previous diagnostic testing included serum biochemistry and hematology, basal cortisol concentration, serum canine pancreatic lipase, cobalamin and folate concentrations, abdominal ultrasound imaging, fecal flotation, and Giardia SNAP test and, for 19/24 dogs, histologic examination of tissues obtained via endoscopy from the stomach, small intestine, and large intestine. Included dogs had either previously failed evidence‐based treatment, such as multiple adequately performed dietary trials, prebiotics, probiotics, immunosuppressive treatment, FMT, or some combination of these, or responded partially or fully to corticosteroids. Included corticosteroid‐responsive dogs required daily maintenance doses of corticosteroids that were associated with marked adverse effects, such as calcinosis cutis (2/5), hypotrichosis and thin skin (3/5), marked muscle loss (2/5) and weight gain (2/5) [20, 21, 24]. Antibiotic trials were generally not performed, consistent with recent recommendations [21, 25]. Dogs with partial response to corticosteroids had experienced a mild decrease in CIBDAI with corticosteroids but continued to have clinical signs that were regarded as cumbersome and deteriorated if treatment was tapered. At least two unsuccessful attempts to taper corticosteroids had been performed in all of these dogs, which were classified as having partially immunosuppressant‐responsive enteropathy (P‐IRE). Exclusion criteria were treatment with antibiotics within 8 weeks before starting cholestyramine or incomplete medical records.

Plasma taurine concentrations were analyzed in a subset of CE and bile acid sequestrant responsive (BASR) dogs during treatment with BAS. The healthy group consisted of 18 staff‐owned dogs from ESAHHS, undergoing fecal donor screening. These dogs had not been treated with antibiotics for a minimum of 6 months and were not receiving any treatments other than ectoparasite prevention and yearly vaccinations.

The CIBDAI was calculated at the baseline visit or retrospectively within 1 month of the baseline visit. The clinical history template at ESAHHS contains all of the anamnestic questions required to calculate CIBDAI. Additionally, fecal consistency was graded from 1 to 7 by the dog owners, using a previously published fecal scoring chart [26]. Dogs were followed from starting BAS until the time of manuscript submission.

### 2.3 Storage and Shipping of Fecal Samples

Baseline fecal samples, collected by the dog owners for diagnostic purposes, were brought to the ESAHHS in connection with scheduled consultations and were stored at −20°C. If the decision to start BAS treatment was made by phone consultation, the dog owners collected a fecal sample, stored it in a refrigerator, and delivered it to the ESAHHS within 24 h of collection, or stored it in a freezer and brought it to the ESAHHS on cool packs in a cool bag at the next scheduled appointment. Additionally, 31 follow‐up fecal samples during treatment with BAS were available from 13 responders. Samples were shipped on dry ice with priority shipment every 6–12 months to the Gastrointestinal Laboratory at the Texas A&M College of Veterinary Medicine and Biochemical Sciences. The condition of the samples was recorded on arrival, and samples then were stored at −80°C. Internal data show good stability of fecal BAs stored at −80°C for 1.5–4 years (Figure S1).

### 2.4 Fecal Bile Acid Analysis

Fecal concentrations of 28 BA (including unconjugated, taurine‐ and glycine‐conjugated, and iso‐ and oxo‐BA) were measured using a previously validated liquid chromatography–tandem mass spectrometry (LC–MS/MS) assay [27]. Primary BA were defined as cholic acid (CA) and chenodeoxycholic acid (CDCA) [28], and 2BA as deoxycholic acid (DCA) and lithocholic acid (LCA), similar to other reports [4, 29, 30, 31]. Deoxycholic acid and LCA are produced by microbial biotransformation of 1BA involving 7α‐dehydroxylation enzymes produced by the bile‐acid‐inducible (*bai*) operon of *P. hiranonis* in dogs [17].

### 2.5 Bacterial Quantitative Polymerase Chain Reaction (qPCR) Analysis

Bacterial qPCR analysis was performed on all fecal samples according to a previously described method [18]. Dysbiosis index (DI) was used to evaluate dysbiosis. This index has been shown to significantly differentiate healthy dogs from dogs with chronic enteropathies and correlate with metagenomic sequencing [18, 32, 33].

### 2.6 Clinical Pathology and Histopathology

Routine serum biochemistry and hematology was performed at the ESAHHS using Indiko Plus (Thermo Fisher Scientific, Gothenburg, Sweden) and ProCyte Dx (IDEXX GmbH Ludwigsburg, Germany), respectively. Serum cholesterol concentration was compared at baseline and during treatment in responders. If multiple follow‐up samples were available from the same dog, the most recent sample was used for comparison.

Serum cobalamin and folate concentrations were analyzed at the Evidensia Specialist Animal Hospital, Strömsholm, Sweden, using an Immulite 2000, and serum specific canine pancreatic lipase was measured at IDEXX BioAnalytics, Kornwestheim, Germany. For plasma taurine concentration, whole blood samples were collected in cold EDTA tubes, which were immediately put in a refrigerator. Plasma was separated the same day, frozen for a minimum of 24 h, and transported in specially designed freeze packs to the Medizinisches Labor Bremen in Germany. Accidentally thawed samples were discarded.

### 2.7 Statistical Analysis

For this retrospective case series of medical records and laboratory data, based on the recommendation of a statistician, no formal statistical comparisons were employed. Data are presented in a descriptive manner.

## 3 Results

### 3.1 Study Population

Twenty‐four dogs with CE and 18 healthy control dogs were included. Age, body weight, sex, breed, body condition score (BCS) and CIBDAI at inclusion are presented in Table 1. Dogs with CE were under treatment for a median of 30 (range, 11–82) months before starting BAS. The main reasons for starting BAS treatment were refractory diarrhea (20/24), abdominal pain, bloating, lip‐smacking, lethargy, borborygmi, and nausea (3/24) and frequent passage of soft feces, urgency, and nightly defecation (1/24). The terms “responders” or CE+BASR (bile acid sequestrant responders) and “non‐responders” or CE−BASR (bile acid sequestrant non‐responders) are used in the remaining text instead of BAD. Non‐responders all had refractory diarrhea. In 12/24 dogs, a second reason for starting BAS was the difficulty in decreasing the corticosteroid dose to an acceptable maintenance dose without recurrence of clinical signs. All dogs had undergone multiple dietary trials before starting BAS, psyllium, or a psyllium‐containing prebiotic and probiotics (Table 1). At inclusion, 21/24 dogs were being treated with corticosteroids, and 20/24 were being treated with second‐line immunosuppressives that were started 75–1151 days before BAS, of which five were receiving treatment with mycophenolate (3–19 mg/kg q24h). Mycophenolate had been added to treat gastrointestinal signs 206–1151 days previously, at a median dosage of 8 mg/kg/day (range, 3–19). This treatment was associated with mild improvement of clinical signs, the possibility to taper corticosteroids slightly, or both, and had not caused worsening of diarrhea in any of the dogs. Previous treatment, discontinued before inclusion, included corticosteroids in 3/24 dogs and alternative second‐line immunosuppressives in 15/24 dogs (Table 1). Regarding phenotype, the population of dogs was fairly heterogeneous. Fourteen of 24 dogs had P‐IRE and six dogs had immunosuppressant‐responsive enteropathy (IRE) but required high doses of corticosteroids, associated with adverse effects described in Section 2. The addition of second‐line immunosuppressives had not allowed tapering of corticosteroids in IRE dogs. Four dogs had non‐responsive enteropathy (NRE), of which one had protein‐losing enteropathy with a serum albumin concentration < 20 g/L on multiple occasions. Results from DI analysis with or without fBA were available in nine dogs when the decision to start treatment with BAS was made, but not in the remaining dogs.

**TABLE 1:** Selected data of healthy dogs and CE dogs responding to versus not responding to BA sequestrants and at inclusion, including previous treatments and diets.

Parameter (range; median or number)	CE+BASR	CE−BASR	Healthy
*n*	16	8	18
Age (years)	3.3–13.3 (7.9)	1.8–8.9 (3.5)	1.0–9.8 (4.3)
BW (kg) at inclusion	6.5–53.3 (23.4)	5.5–28.5 (16.7)	6.7–31.1 (14.4)
BCS at inclusion (/9)	3–6 (5)	3–7 (5)	4–6 (5)
Breeds (amount) > 1 dog/group	Havanese (2), Labrador r. (2), Rottweiler (2)	English Springer Spaniel (2), Whippet (2)	Mixed br. (3), Shetland Sheepdog (2), Whippet (2)
Sex (M/MN/F/FN)	5/6/3/2	3/2/2/1	4/3/8/3
Phenotype	P‐IRE (10), IRE (4), NRE (2)	P‐IRE (4), IRE (2), NRE (1), NRE + PLE (1)	H (18)
CIBDAI at inclusion	4–13 (6.5)	4–8 (5)	1–2 (1)
Fecal score (/7)	1–7 (5)	4–6 (4.5)	1–3 (2)
Serum albumin (g/L); RI 29–39	24–42 (31)	25–36 (31)	31–39 (33)
Albumin 24–28 g/L	5/16 (31%)	1/8 (13%)	0/18
Serum total protein (g/L); RI 59–75	43–73 (63)	42–77 (60)	n/a
Total protein below RI	1/16 (6%)	2/8 (25%)	n/a
Serum cholesterol (mg/dL); RI 159–282	124–371 (260)	151–398 (273)	n/a
Cholesterol below RI	1/16 (6%)	1/8 (13%)	n/a
Serum ALT activity (U/L); RI 18–78	18–192 (57)	30–288 (51)	n/a
Hematocrit (%), RI 37–62	29–53 (42)	34–63 (44)	n/a
Serum cobalamin (ng/L); RI 251–908	267–1000 (578)	373–913 (630)	474–878 (643)
Serum folate (μg/L); RI 6.6–19.8	4.4–23.8 (15.7)	11–23.8 (18.8)	n/a
Serum cPL (μg/L); RI 0–200	44–2000 (115)	32–208 (55)	n/a
Diet at inclusion			
KD; maintenance diet	n/a	1	16
KD: “Intestinal”	2	n/a	1
KD; single protein	3	2	1
KD; hydrolyzed	8	5	n/a
Home‐cooked	3^a^	n/a	n/a
Previous dietary trials/dog	3–7 (5)	3–9 (5)	n/a
Type of previous diet	HY (23), SP (20), I (15), HC (5), HF (4), LF (3)	HY(13), SP (18), I (7), HC (4), HF (3)	n/a
Treatment at inclusion			
Corticosteroids	14	7	n/a
Started before inclusion (days)	471–2496 (1228)	267–1090 (639)	n/a
Second line immunosuppressives	CSA (8), MMF (5)	CHL (3), CSA (3)	n/a
Started before inclusion (days)	75–1151 (396)	60–670 (209)	n/a
Cobalamin supplement	5/16	2/8	n/a
Started before inclusion (days)	180–1156 (609)	214–275	n/a
Folate supplement	1/16	0	n/a
Started before inclusion (days)	833	n/a	n/a
Prebiotics	3	3	n/a
Probiotics	3	6	n/a
Previous treatment			
Corticosteroids	2	1	n/a
Second line immunosuppressives	AZA (2), CSA (5), MMF (2)	AZA (2), MMF (4)	n/a
Prebiotic trial (psyllium)	16	8	n/a
Probiotic trial	14	8	n/a

Abbreviations: +BASR, responsive to bile acid sequestrants; 2nd, second; AZA, azathioprine; BA, bile acid; −BASR, non‐responsive to bile acid sequestrants; BCS, body condition score; BW, body weight; CE, chronic enteropathy; CHL, chlorambucil; CIBDAI, Canine inflammatory bowel disease index; cPL, canine pancreatic lipase; CSA, Cyclosporine A; H, healthy; HC, home‐cooked balanced diet; HF, high‐fiber diet; HY, hydrolyzed protein diet; I, intestinal diet; IRE, immunosuppressive‐responsive enteropathy; KD, kibble diet; LF, low fat diet; MMF, mycophenolate mofetil; n/a, not applicable; NRE, non‐responsive enteropathy; P‐IRE, partially immunosuppressive‐responsive enteropathy; PLE, protein‐losing enteropathy, defined as having serum albumin concentrations below 20 g/L on multiple occasions; RI, reference interval; SP, single protein.

^a^ Balanced according to AAFCO norms.

### 3.2 Response to Treatment With BAS—CIBDAI and Additional Variables

**FIGURE 1:**
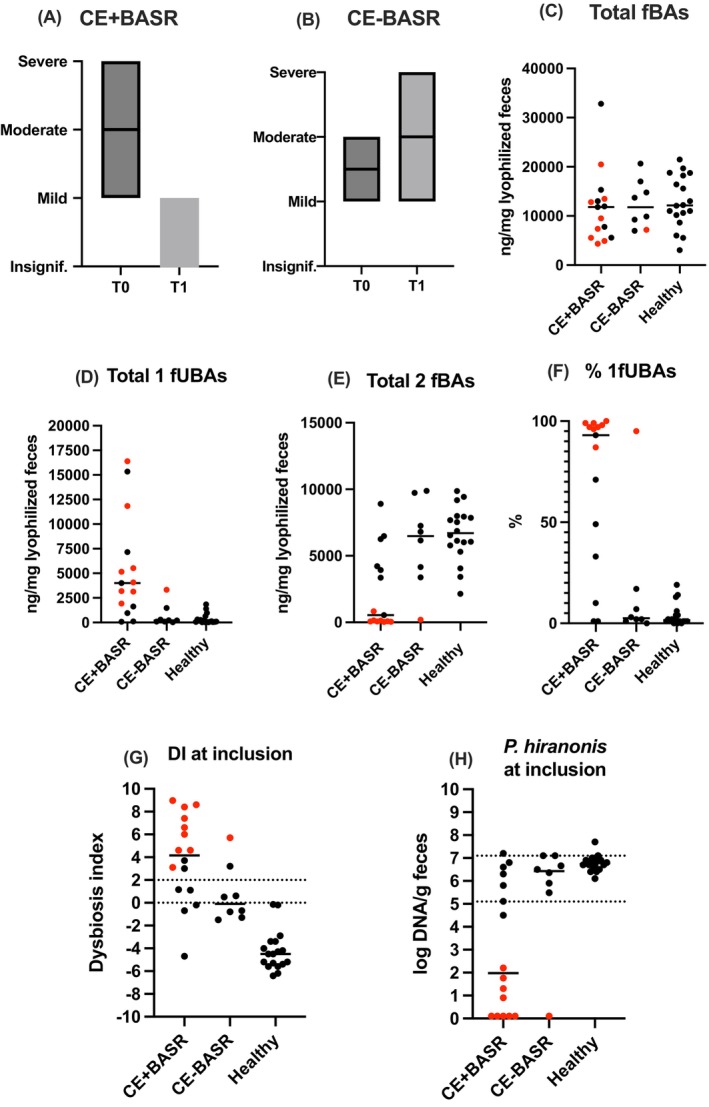
CIBDAI categories, fecal BA, DI and abundance of *Peptacetobacter hiranonis*. CIBDAI categories at baseline and T1 in dogs with CE responding to (A) versus not responding to (B) BA sequestrants. Fecal BA at inclusion (C–F). DI (G) and abundance of *P. hiranonis* (H) at inclusion. (C) total fBA represent a sum of all BA measured, (D) total 1fBA represent conjugated and unconjugated cholic acid and chenodeoxycholic acid, (E) total 2fBA represent conjugated and unconjugated deoxycholic acid and lithocholic acid, (F) represent the unconjugated 1fBA as a percentage of unconjugated fBA. Bars represent the median. Red dots represent dogs with log10 abundance of *P. hiranonis* < 4.5. Gray field represent reference interval. BA, bile acid; +BASR, responding to bile acid sequestrants; CE, chronic enteropathy; +BASR, non‐responsive to bile acid sequestrants; CIBDAI, canine inflammatory bowel disease activity index; DI, dysbiosis index; fBA, fecal bile acids; fUBA, fecal unconjugated bile acids; T0, baseline; T1, 21–35 days after starting BAS.

The first follow‐up time point was 21–35 days after starting off‐label treatment with BAS. Multiple follow‐up time points were available from all responders. Sixteen of 24 dogs responded to BAS, with a decrease in CIBDAI (Table 2) and CIBDAI category, from severe to mild, moderate to mild, or insignificant, and mild to insignificant (Figure 1). Fecal scores decreased from a median of 5 (1–7 out of 7) to a median of 2.5 (2–5 out of 7) during treatment in CE+BASR dogs. Three additional dogs showed improvement of other clinical signs not accounted for in the CIBDAI, such as nocturnal defecation or abdominal pain, bloating, or a swollen abdomen. In nine of 14 CE+BASR dogs treated with corticosteroids at inclusion, the maintenance dose of corticosteroids could be tapered by 33%–67%, which had not been possible before starting BAS. Non‐responders had no decrease in CIBDAI category or fecal scores (Figure 1 and Table 2).

**TABLE 2:** Selected data in CE dogs responding to versus not responding to BA sequestrants.

Parameter (range; median or number)	CE+BASR T0	CE+BASR T1	CE−BASR T0	CE−BASR T1
CIBDAI	4–13 (6.5)	2–5 (3)	4–8 (5)	4–9 (6.5)
Fecal score (/7)	1–7 (5)	2–5 (2.5)	4–6 (4.5)	4–7 (5)
Serum albumin (g/L); RI 29–39	24–42 (31)	24–35 (32)	25–36 (31)	
Albumin 24–28 g/L	5/16 (31%)	4/16 (25%)^a^	1/8 (13%)	n/a
Serum cholesterol (mg/dL); RI 159–282	104–371 (260)	166–356^a^ (288)	151–398 (273)	n/a
Plasma taurine (μmol/L); RI 44–224	n/a	16–376^a^ (120)	n/a	n/a
Euthanasia for GI reasons	n/a	4/16 (25%)^b^	n/a	4/8 (50%)^b^

Abbreviations: +BASR, responsive to bile acid sequestrants; −BASR, non‐responsive to bile acid sequestrants; CE, chronic enteropathy; CIBDAI, Canine inflammatory bowel disease index; T0, baseline; T1, 21–35 days after starting BAS.

^a^ Latest sample collected.

^b^ Until time of writing.

### 3.3 BA Sequestrants and Adverse Effects

The median (range) starting dose of cholestyramine (Kolestyramin^1^ Alternova, Orifarm Generics AB, Stockholm, Sweden) was 52 (22–79) mg/kg q24h for 5–7 days, which was gradually increased weekly over 3–4 weeks to a maximum dose of 64 (44–133) mg/kg q12h, unless marked adverse gastrointestinal effects occurred. This gradual increase and dose range mirror recommendations in people [34, 35]. Owners were instructed to give any concurrent medication one hour before BAS or 4–6 h later, as recommended in people [34, 35]. Seven dogs were treated with second‐generation BAS: 6/7 dogs received colesevelam (Cholestagel^2^, Cheplapharm, Greifswald, Germany), and 1/7 colestipol (Lestid^3^, Pfizer, Stockholm, Sweden; no longer available). This decision was based on the lack of clinical response to cholestyramine in 4/7 dogs, palatability issues in 1/7 dogs, and a shortage of cholestyramine in 2/7 dogs. Colesevelam was given at a starting dosage of 5–24 mg/kg (median, 11) q24h, which was gradually increased to 7 to 24 mg/kg (median, 11) q12h. There was limited ability to titrate the colesevelam dose based on the available tablet formulation. Colestipol was given at a dosage of 26 mg/kg q24h. Of the four dogs previously non‐responsive to cholestyramine, one responded to colestipol and one to colesevelam. These two dogs thus were classified as BAS responders. The dog with palatability issues on cholestyramine had a better clinical response to colesevelam than cholestyramine regarding fecal quality and frequency. Adverse effects of cholestyramine were seen in 4/16 responders and 5/8 non‐responders. Two responders vomited on two to three occasions during the first week of treatment, one had increased flatulence, and one dog developed diarrhea, bloating, and signs of abdominal pain on cholestyramine, but tolerated second‐generation BAS well. One dog treated with colesevelam developed melena and worsening of diarrhea at a starting dosage of 20 mg/kg q24h, but later tolerated a starting dose of 5 mg/kg q 24h. Colesevelam then was gradually increased to 15 mg/kg q24h without adverse effects, but with markedly improved fecal quality and decreased flatulence. The only dog treated with colestipol responded clinically to a dosage of 26 mg/kg q24h without adverse effects, but developed diarrhea and a distended abdomen when the dosage was increased to q12h. The dosage then was decreased to q24h again, which had a good effect on clinical signs.

In non‐responders, 4/8 dogs showed worsening of diarrhea and 1/8 dogs developed vomiting and diarrhea. In 3/8 non‐responders, adverse effects occurred when the dosage was increased to q12h. Subsequently, the dosage was decreased to q24h again. Two of the non‐responders had BAS discontinued within 14 days of treatment because of adverse events. Remaining non‐responders continued BAS for a minimum of 4 weeks, without any clinical improvement.

### 3.4 Fecal Bile Acid Profiles at Baseline and Follow‐Up

Samples for baseline fBA were analyzed in 23/24 diseased dogs and 18 healthy dogs (Table 3).

**TABLE 3:** Fecal BA in CE+BASR, CE−BASR and healthy dogs.

ng/mg fecal dry matter or %	CE+BASR		CE−BASR		Healthy	
	Median	Range	Median	Range	Median	Range
Total BA	11 881	4362–32 816	11 798	6992–20 642	12 147	3049–21 486
% UBA of total BA	99	94–100	99	98–100	99	98–100
Total 1BA	4013	73–16 388	216	25–3321	106	20–1846
% 1 UBA of total UBA	93	1–100	3	0–95	2	0–19
Total 2BA	540	24–8902	6480	189–9883	6780	2138–9870
Unconjugated BA (UBA)						
Cholic acid (CA)	3718	32–14 236	148	16–2812	62	10–1699
3oxo‐CA	52.1	1–205.8	12.9	0.4–50.6	1.6	0.3–45.9
iso‐CA	106.3	6.5–832.7	27.1	1.7–85.1	14.9	1.7–117.2
Chenodeoxycholic acid (CDCA)	269.8	19.5–2453	59.1	7–481.7	34.6	4.6–312.3
3oxo‐CDCA	8.0	2.0–45.0	2.5	1.0–14.0	3.0	1.0–14.0
Deoxycholic acid (DCA)	481	29–8186	5421	172–9361	5183	1593–8419
3oxo‐DCA	6	1–851	582.5	5–4839	682	50–2475
7oxo‐DCA	1903	8–8232	119	13–2151	53	4–952
Lithocholic acid (LCA)	8	4–844	605.5	11–1474	1213	289–3728
12oxo‐LCA	12	0–2651	781.5	9–4839	1486	90–5079
3,7,12oxo‐LCA	0.2	0.0–0.9	0.2	0.0–0.5	0.2	0–0.6
7,12oxo‐LCA	6.8	0.4–205.8	4.2	0.4–19	2.7	0.3–21.9
7oxo‐LCA	90.9	8.8–541.9	20.1	4.4–278	31.8	2.8–197.4
Allo‐LCA	0.0	0.0–0.2	0.0	0.0–0.1	0.0	0.0–0.0
Hyocholic acid (HCA)	0.6	0.2–3.3	0.9	0.2–1.6	1.5	0.3–4.6
Hyodeoxycholic acid (HDCA)	229	1–2205	1804	95–4550	2298	355–7243
Ursocholic acid (UCA)	48.2	0.1–1137	15.2	1.8–810	13.7	2.5–171.4
Ursodeoxycholic acid (UDCA)	22.6	0.6–433	14.7	1.8–125.9	15	5.6–68.7
Conjugated BA						
Glycocholic acid (GCA)	5	0.2–19.3	0.7	0–3.2	0.6	0.1–4.6
Glycochenodeoxycholic acid (GCDCA)	0.4	0.2–1.7	0.3	0.0–0.8	0.7	0.0–3.0
Glycodeoxycholic acid (GDCA)	0.2	0.0–5.5	4.3	0.1–16.8	3.4	0.4–22.4
Glycolithocolic acid (GLCA)	0.0	0.0–0.7	0.4	0.1–1.9	0.7	0.2–4.2
Glycoursodeoxicholic acid (GUDCA)	0.0	0.0–0.5	0.1	0.0–0.3	0.0	0.0–0.1
Taurocholic acid (TCA)	27.1	2.9–171.8	4.4	1.1–20.9	6.6	0.6–29.3
Taurochenodeoxycholic acid (TCDCA)	8.6	0.2–43.3	2.1	0.2–21.5	2.1	0.1–6.8
Taurodeoxycholic acid (TDCA)	1.4	0.0–40.7	5.6	0.7–121.5	20.0	1.5–122.6
Taurolithocolic acid (TLCA)	0.5	0.0–2.1	1.2	0.1–8	2.0	0.1–16.5
Tauroursodeoxicholic acid (TUDCA)	1.7	0.1–18	0.2	0–5.8	0.1	0.0–0.7

*Note:* Total BA, sum of all 28 BA; Total 1BA, sum of conjugated and unconjugated cholic acid and chenodeoxycholic acid; Total 2BA, conjugated and unconjugated deoxycholic acid and lithocholic acid; % UBA of total BA, % unconjugated BA of all 28 BA in conjugated or unconjugated form; % 1 UBA of total UBA, % unconjugated cholic acid and chenodeoxycholic acid of all 28 unconjugated BA.

Abbreviations: BA, bile acids; CE+BASR, chronic enteropathy responding to bile acid sequestrants; CE−BASR, chronic enteropathy non‐responsive to bile acid sequestrants; *p*, *p* value; UBA, unconjugated bile acid.

Unconjugated fBA represented 94%–100% of the total fBA in both groups (Table 3).

**FIGURE 2:**
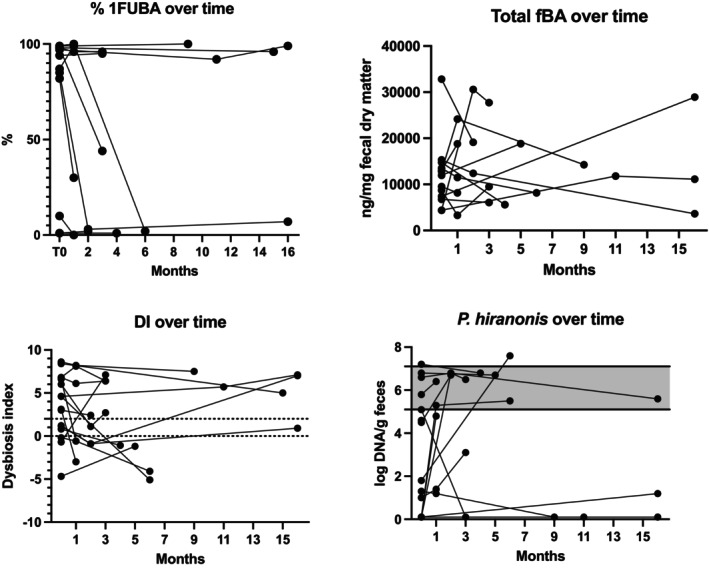
Fecal unconjugated bile acids, total fecal bile acids, dysbiosis index and abundance of *Peptacetobacter hiranonis* over time in CE dogs responding to BAS. % 1FUBA represent the unconjugated 1fBA as a percentage of unconjugated fBA. Total fBA represent the sum of all BA measured. Gray field represent reference interval. 1fBA, primary fecal bile acids; +BASR, responding to bile acid sequestrants; −BASR, non‐responsive to bile acid sequestrants; CE, chronic enteropathy; DI, dysbiosis index; fBA, fecal bile acids; fUBA, fecal unconjugated bile acids.

In healthy dogs and CE‐BASR dogs, the majority of fBA at baseline were 2BA, consistent with previous studies (Figure 1) [5, 6, 7]. The amount of 2BA was lower in CE+BASR dogs compared with healthy and CE‐BASR dogs. Dogs responding to BAS had increased abundance of total f1BA and % f1BA as compared with non‐responders and healthy dogs (Figure 1) at baseline. Responders had (mean [95% confidence interval (CI)]) increased percentage (69% [47–90]) of unconjugated 1BA versus non‐responders (16% [−11 to 43]) and healthy dogs (4% [1–7]), as well as increased total amount of f1BA (5368 [2481–8256]) versus non‐responders (717 [−246 to 1680]) and healthy dogs (355 [93–617]). Follow‐up fecal samples, collected at various time points, from CE+BASR dogs during treatment were available from 13 dogs. Eleven of 13 dogs had the first follow‐up sample collected 1–4 months after starting BAS. Results are presented with a timeline in Figure 2.

### 3.5 Microbiota qPCR Analysis

Fecal samples for DI at baseline were available from all dogs. Median [range] DI in CE+BASR dogs was 4.2 [−4.7 to 9.0], −0.1 [−1.5 to 5.7] in CE‐BASR dogs, and −4.5 [−6.4 to −0.2] in healthy dogs (Figure 1). Median [range] abundance of *P. hiranonis* log DNA was lower in responders (2.0 [0.1–7.2]) versus non‐responders (6.4 [0.1–7.0]) and healthy dogs (6.7 [6.1–7.7]; Figure 1). Eleven of 16 CE+BASR dogs had depletion of *P. hiranonis* at baseline compared with 1/8 CE−BASR dogs and 0/18 healthy dogs. Follow‐up samples during treatment with BAS were available from 14 responders (Figure 2).

### 3.6 Serum Cholesterol and Plasma Taurine Concentrations

Serum cholesterol concentrations at baseline and during treatment with BAS were available from 10 responders (Table 2). No marked difference in serum cholesterol concentration was observed at baseline as compared to follow‐up. Two additional dogs lacked baseline samples but had serum cholesterol concentrations within the reference interval (RI) during treatment. Only 1 of 30 serum cholesterol concentrations from 12 dogs during BAS treatment was subnormal. This dog had serum cholesterol concentrations within the RI in several samples collected before and after that time point.

Plasma taurine concentrations during treatment were available from 9 CE+BASR dogs (Table 2). Samples were collected a median of 9 (range, 4–34) months after starting treatment with BAS. Follow‐up plasma taurine concentrations were within or above the RI in 8/9 dogs (RI, 44–224 μmol/L). One asymptomatic dog had a subnormal plasma taurine concentration of 16 μmol/L 7 months after starting cholestyramine. Serum cholesterol concentration was unaltered compared to baseline at this time point. Treatment with cholestyramine was stopped and taurine supplementation was initiated. At follow‐up, the plasma taurine concentration had normalized. Three other dogs had second follow‐up taurine samples. None of these samples had lower serum taurine concentrations than the first follow‐up samples.

### 3.7 Long‐Term Follow Up

Euthanasia because of NRE or severe flare‐up of CE was elected in 4/8 CE−BASR dogs and 4/16 CE+BASR dogs. Seven BAS responders had been euthanized for non‐GI disorders. These dogs could be maintained with 33%–67% lower dosages of corticosteroids than before starting BAS and were clinically more stable until the time of death. The CE+BASR dogs alive at the time of manuscript preparation had a median (range) CIBDAI of 2.5 (2–3) at the last follow‐up visit within 1–6 months of manuscript preparation, which is lower than their corresponding baseline median (range) CIBDAI of 5 (4–8). Additional clinical long‐term follow‐up information is available in Data S4.

## 4 Discussion

In this retrospective study, we report the clinical picture and fecal profile in 24 dogs with non‐responsive or partially immunosuppressive‐responsive enteropathy, or CE dogs requiring high dosages of corticosteroids, responding to versus not responding to BAS. Furthermore, we report the long‐term clinical response and adverse effects of treatment with BAS. Sixteen of 24 dogs responded to BAS, with improved fecal scores and decreased CIBDAI category. At baseline, CE+BASR dogs had increased amounts and percentages of f1BA, but not total fBA, compared with non‐responders and healthy dogs. In addition, dogs with CE+BASR had higher DI and lower abundance of *P. hiranonis* compared with CE−BASR and healthy dogs.

Fecal 1BA differed in CE+BASR from CE−BASR and healthy dogs. The majority of the CE+BASR dogs had depletion of *P. hiranonis*, a bacterial species that carries the bile acid‐inducible (*bai*) operon. The *bai* operon encodes for enzymes that are capable of both deconjugation and dehydroxylation of f1BA into f2BA. A positive correlation among the abundance of *P. hiranonis*, the *bai* operon, and f2BA recently has been shown in dogs [4]. Two separate studies have shown that conversion from f1BA into f2BA requires a log10 abundance of *P. hiranonis* above a threshold of 4.5 [3, 4]. This threshold is in agreement with our findings, where all dogs with depletion of *P. hiranonis* had very low amounts of f2BA compared with CE‐BASR and healthy dogs. Hence, the abundance of *P. hiranonis* is a good predictor of BA conversion.

Four CE+BASR dogs had a high percentage of f1BA compared with healthy and CE−BASR dogs, despite normal abundance of *P. hiranonis*. This finding could result from decreased absorptive capacity of the ileum, disruption of the negative feedback loop in BA synthesis, or both, resulting in overproduction of 1BA [10]. Three additional CE+BASR dogs with chronic diarrhea had neither dysbiosis nor a high amount or percentage of f1BA but responded well to BAS. These dogs may have had other alterations of normal BA cycling or decreased intestinal transit time.

The increased amount of 7oxo‐deoxycholic acid (7oxo‐DCA) and 7oxo‐lithocolic acid (7oxo‐LCA) in some of the CE+BASR dogs may seem surprising, because deoxycholic acid and lithocholic acid are both 2BA. However, the transformation of cholic acid and chenodeoxycholic acid to 7oxo‐DCA and 7oxo‐LCA, respectively, does not involve the *bai* operon, but rather uses the enzyme 7α‐hydroxysteroid dehydrogenase (HSDH) [29, 36, 37]. Multiple bacterial taxa, including *Escherichia coli* and several *Clostridium* spp., carry HSDH genes [29]. Although f1BA can be transformed through oxidation and epimerization by different forms of HSDH, this change only accounts for a small fraction of 1BA conversion compared with 7α‐dehydroxylation via the *bai* operon in people [29]. In the CE+BASR dogs, a large amount of 1BA is present in the feces. This situation, combined with depletion of *P. hiranonis,* potentially could lead to increased utilization of BA transformation via the 7α‐HSDH pathway and corresponding increase in 7oxo‐LCA and 7oxo‐DCA.

The increased amount of f1BA, but not total fBA observed in our study, also has been found in people with IBD [38]. However, studies in people with irritable bowel syndrome with diarrhea (IBS‐D) have shown an increase in the total amount of fBA in BAD [39, 40, 41]. It is possible that alterations in fBA are related to which type of BAD affects the patient. Type 1 includes patients with ileal dysfunction (Crohn's disease) or absent ileum; type 2 is primary or idiopathic BAD in patients lacking intestinal morphological abnormalities, such as IBS‐D; and type 3 is BAD secondary to miscellaneous GI disorders, including microscopic colitis, small intestinal bacterial overgrowth, and others. In our observational study, all CE+BASR dogs appeared to be affected as a consequence of CE, with or without intestinal dysbiosis. Flare‐ups of diarrhea, necessitating dose increases or changes of immunomodulatory treatment, still occurred in several dogs during treatment with BAS. In our experience, increasing the dose of BAS usually was not sufficient to decrease disease activity during a flare‐up in these dogs. The often persistently abnormal unconjugated f1BA, DI, and abundance of *P. hiranonis* in CE+BASR dogs over time suggest that the underlying disease is still present, but clinical signs are dampened by BAS in combination with other treatments.

The CE‐BASR dogs had less marked dysbiosis than the responders. Not all CE dogs have dysbiosis, and we hypothesize that the CE‐BASR dogs have a different phenotype than the CE+BASR dogs.

The ability to decrease the maintenance dose of corticosteroid by 33%–67% after starting BAS treatment in 9/14 CE+BASR dogs is encouraging. Four of these dogs had IRE with previously described marked adverse corticosteroid effects. These adverse effects improved after tapering corticosteroids in these dogs. For the remaining five dogs, two became less heat intolerant, one became more active, one stopped having recurrent urinary tract infections, and one overweight dog lost weight. These findings are consistent with a recent study on BAS treatment in people with microscopic colitis, in which 37 of 45 patients could decrease budesonide after starting BAS [42]. Studies in healthy people and rats have shown that corticosteroid treatment increases the expression of ASBT up to 30% [43, 44]. This increase may be a contributing factor to why it was not possible to decrease corticosteroids, despite the addition of second or third line immunosuppressives, in CE+BASR dogs until treatment with BAS was initiated. Other possible explanations include that high doses of corticosteroids were needed to combat inflammation and that binding an excessive amount of 1BA had an anti‐inflammatory effect that enabled tapering of corticosteroids.

Bile acid sequestrants are mainly used for the treatment of primary hypercholesterolemia in people [45]. Treatment with BAS for chronic diarrhea thus poses a risk of causing hypocholesterolemia. The maintenance doses of BAS used in our study were too low to induce hypocholesterolemia, except for a transient, short‐lasting decrease in one dog on one occasion after cholestyramine had been increased because of a flare‐up. Because the dogs in our study did not develop persistent hypocholesterolemia, we suspect that the risk of developing deficiencies in fat‐soluble vitamins over time is likely low.

Treatment with BAS further poses a risk of taurine deficiency, because cholestyramine in people has a higher affinity for BA conjugated with taurine than for those conjugated with glycine [46]. One asymptomatic dog in our study was diagnosed with taurine deficiency based on a plasma sample collected after 7 months of BAS treatment. Treatment was stopped, and supplementation with taurine started. Taurine deficiency can contribute to dilated cardiomyopathy and premature death in dogs [47]. Consequently, it is crucial to monitor plasma taurine concentrations in dogs being treated with BAS or to supplement all dogs treated with BAS with taurine, although 8 of 9 dogs with a known taurine status had normal plasma concentrations. Additional studies are needed to assess the prevalence of iatrogenic taurine deficiency in dogs treated with BAS, especially because taurine deficiency may not be detected by plasma taurine concentrations unless the deficiency is severe [48, 49].

The clinical response in the CE+BASR dogs varied from a marked decrease in frequency of defecation and improvement in fecal quality to slightly improved frequency of defecation and improved fecal scores or decreased urgency. This variation may correlate with the severity of BAM consistent with data from people. In a systematic review, a dose–response relationship between severity of BAM and clinical response to BAS was shown [50]. Response to cholestyramine was seen in 96% of the patients with severe BAM, based on the 75‐selenium homocholic acid taurine test, in 80% of patients with moderate BAM and in 70% of patients with mild BAM.

In our observational study, 7/16 dogs responded to second generation BAS, such as colesevelam (6/16) or colestipol (1/16). Two of the dogs previously had not responded to cholestyramine. Colesevelam has reported efficacy in people with BAD who do not respond to cholestyramine or in patients who discontinue cholestyramine because of adverse effects [35, 51]. Cholestyramine is associated with a high rate of discontinuation in people because of adverse events, even in patients who have clinical improvement of diarrhea [35, 52, 53]. A very recent prospective randomized trial showed that colesevelam was well tolerated and superior to placebo at inducing remission of BAD in people [54]. Very little information is available on colesevelam in dogs. In a study in rats and dogs, using radioactively labeled colesevelam, all of the colesevelam was excreted in feces in both species after 28 days of treatment [55]. An unpublished 28‐day toxicity study of colesevelam in rats and dogs previously was performed by the same group [55]. No GI lesions were found, in contrast to another study in rats that showed villous atrophy and colonic cell injury after cholestyramine or colestipol treatment [56]. A document from the European Medicines Agency's scientific evaluation of colesevelam for human consumption is available online and contains data from dogs [57]. Colesevelam dosages of 300 mg/kg or higher q24h lowered serum cholesterol concentrations in dogs. Toxicity studies lasting 1 year suggested no observed adverse effects at a dosage of 200 mg/kg q24h, and a lowest observed adverse effect dosage of 600 mg/kg q24h. These dosages of colesevelam are markedly higher than the 7–24 mg/kg q12h used in our observational case series. However, colesevelam at a starting dosage of 20 mg/kg was associated with abdominal pain, melena, and mucoid feces in one dog, which required discontinuation of colesevelam and restarting cholestyramine. One year later, the dog refused the cholestyramine, and colesevelam was started at a lower dosage and slowly increased to 15 mg/kg q24h without adverse effects. Furthermore, fecal quality and frequency were improved compared with during cholestyramine treatment.

Normalization of fBA dysmetabolism can occur in some dogs with food‐responsive enteropathy and steroid‐responsive enteropathy after treatment with diet or corticosteroids [5, 17]. Dogs suspected to have BA dysmetabolism always should be treated with conventional treatment protocols first, including targeted nutritional interventions such as supplemental fiber (e.g., psyllium) [20, 21]. Psyllium is known to bind fBA and induce expression of genes mediating BA secretion, preventing reabsorption and leading to increased excretion of fBA, and thus acting like a less potent BAS [58, 59]. All CE dogs in our study had been treated using multiple dietary trials, psyllium, and immunomodulatory treatments before BAS. Thus, persistent clinical signs of CE, persistently increased DI, f1BA, depletion of *P. hiranonis*, or some combination of these, despite multiple dietary trials, psyllium, and medical treatments are possible indications for considering BAS.

In the CE+BASR group, 3/16 dogs had bloating, lethargy, and abdominal pain as the main clinical signs and only occasional diarrhea. These clinical signs markedly improved with BAS treatment. Similar results have been reported in people with BAM, with subsequent improvement in abdominal pain, flatulence, lethargy, and bloating noted during treatment with BAS [60].

Our study had several limitations, mostly related to its retrospective design and small sample size. Neither samples for cholesterol and taurine determination nor long‐term follow‐up fecal samples were available from all CE+BASR dogs. The time point for obtaining follow‐up samples also varied. Furthermore, the decision to start BAS was influenced by results from DI or fBA or both in 9/24 CE dogs. Consequently, this population may not represent a general population of dogs with IRE, NRE, or P‐IRE. Additionally, concurrent treatments were not standardized, and groups were heterogeneous. Multiple treatments were given in parallel with BAS, without a wash‐out period. However, the responders had been on the same medical treatment for 75–1151 (median, 396) days before starting BAS, and diet was not changed for a minimum of 4 months, making clinical improvement more likely related to BAS than a late onset effect of concurrent treatment.

Despite these limitations, the results of our study may be useful when designing prospective studies on treatment using BAS in CE dogs partially or completely refractory to other treatments, or requiring high doses of corticosteroids, and having a suspicion of BAM. Bile acid sequestrants target a previously rarely addressed pathophysiology in dogs with CE. When more data is available, the addition of BAS in dogs with refractory CE may contribute to decreasing the number of dogs euthanized for NRE or decreasing disease activity in P‐IRE dogs.

## Disclosure

The authors declare no off‐label use of antimicrobials.

## Ethics Statement

The authors declare no institutional animal care and use committee or other approval was needed. The authors declare human ethics approval was not needed.

## Conflicts of Interest

Linda Toresson, Amanda B. Blake, Chi‐Hsuan Sung, M. Katherine Tolbert, and Jan S. Suchodolski work for the Gastrointestinal Laboratory at Texas A&M University that analyzes dysbiosis index on a fee for service basis. The other authors declare no conflicts of interest.

## Supporting information


**Data S1:** 1A. Histopathologic diagnosis in 12 CE dogs responding to bile acid sequestrants. All biopsies were retrieved endoscopically. Biopses from the small intestine were not available in 1 out of 12 dogs. Biopsies were analyzed according to the guidelines from the World Small Animal Veterinary Association Gastrointestinal Standardization Group.
**Data S2:** Commercial diets at inclusion in 13 CE dogs responding to bile acid sequestrants (CE+BASR) and 8 CE dogs not responding to bile acid sequestrants (CE−BASR). Three dogs responding to bile acid sequestrants were fed a balanced home‐cooked diet.
**Data S3:**. Stability of percentage of fecal unconjugated primary bile acids over time in fecal samples stored in −80°C. Samples stored for 1.5–4 years (median 2.5).

**Data S4:** Long‐term follow up data of CE dogs treated with BAS.

## Endnotes

^1^
Kolestyramin Alternova (4 g), Orifarm Generics, Odense, Denmark.

^2^
Cholestagel (625 mg), Cheplapharm, Greifswald, Germany.

^3^
Lestid (1 g), Pfizer, Sollentuna, Sweden.
