# Supplementary material for: Fecal and Clinical Profiles of Dogs With Chronic Enteropathies Treated With Bile Acid Sequestrants for 5–47 Months: A Retrospective Case Series
Source: J Vet Intern Med. 2025 Aug 20;39(5):e70206. doi: 10.1111/jvim.70206 (PMC12365994; doi:10.1111/jvim.70206)
Supplement: Supplementary file 1 — Data S1: 1A. Histopathologic diagnosis in 12 CE dogs responding to bile acid sequestrants. All biopsies were retrieved endoscopically. Biopses from the small intestine were not available in 1 out of 12 dogs. Biopsies were analyzed according to the guidelines from the World Small Animal Veterinary Association Gastrointestinal Standardization Group. Data S2: Commercial diets at inclusion in 13 CE dogs responding to bile acid sequestrants (CE+BASR) and 8 CE dogs not responding to bile acid sequestrants (CE−BASR). Three dogs responding to bile acid sequestrants were fed a balanced home‐cooked diet. Data S3:. Stability of percentage of fecal unconjugated primary bile acids over time in fecal samples stored in −80°C. Samples stored for 1.5–4 years (median 2.5). [file JVIM-39-e70206-s002.docx]

**SUPPLEMENTARY MATERIAL 1.**

1A. Histopathologic diagnosis in 12 CE dogs responding to bile acid sequestrants. All biopsies were retrieved endoscopically. Biopses from the small intestine were not available in 1 out of 12 dogs. Biopsies were analyzed according to the guidelines from the World Small Animal Veterinary Association Gastrointestinal Standardization Group

| **Location** | **Histopathological findings** | **Severity** | **n** |
| --- | --- | --- | --- |
| Stomach | LP gastritis | Mild | 5 |
|  |  | Moderate | 2 |
|  | Eosinophilic gastritis | Mild | 1 |
|  | LP and eosinophilic gastritis | Mild | 1 |
|  | Mixed cell type (eosinophils/LP /neutrophils) | Mild | 1 |
|  | Normal stomach | N/A | 2 |
| Small intestine | LP enteritis | Mild | 2 |
|  |  | Moderate | 5 |
|  | LP and eosinophilic enteritis  Eosinophilic enteritis  Normal small intestine | Mild  Moderate  N/A | 1  2  1 |
| Large intestine | LP colitis | Mild | 4 |
|  |  | Moderate | 2 |
|  | LP colitis with erosions | Mild | 1 |
|  |  | Moderate | 1 |
|  | LP and eosinophilic colitis | Moderate | 1 |
|  | Eosinophilic colitis | Moderate | 1 |
|  | Normal large intestine | N/A | 2 |

Abbreviations: LP, lymphocytic-plasmacytic; n/a, not applicable

1B. Histopathologic diagnosis in 7 CE dogs not responding to bile acid sequestrants. All biopsies were retrieved endoscopically. Biopses from the small intestine were not available in 2 out of 7 dogs, and biopsies from the colon were not available in 1 out of 7 dogs. Biopsies were analyzed according to the guidelines from the World Small Animal Veterinary Association Gastrointestinal Standardization Group

| **Location** | **Histopathological findings** | **Severity** | **n** |
| --- | --- | --- | --- |
| Stomach | LP gastritis | Mild | 1 |
|  |  | Moderate | 1 |
|  | LP and eosinophilic gastritis with erosions and fibrosis | Moderate | 1 |
|  | LP and eosinophilic gastritis | Moderate | 1 |
|  | LP gastritis with moderate fibrosis | Mild | 1 |
|  |  | Moderate | 1 |
|  | Normal stomach | N/A | 1 |
| Small intestine | LP enteritis | Moderate | 3 |
|  | LP enteritis with erosions | Severe | 1 |
|  | Normal duodenum |  | 1 |
| Large intestine | LP colitis | Mild | 3 |
|  | LP and eosinophilic colitis with erosions | Moderate | 1 |
|  | Normal large intestine | N/A | 2 |

Abbreviations: LP, lymphocytic-plasmacytic; n/a, not applicable

**SUPPLEMENTARY MATERIAL 2.** Commercial diets at inclusion in 13 CE dogs responding to bile acid sequestrants (CE+BASR) and 8 CE dogs not responding to bile acid sequestrants (CE-BASR). Three dogs responding to bile acid sequestrants were fed a balanced home-cooked diet.

|  | CE+BASR | CE-BASR |
| --- | --- | --- |
| KD; maintenance diet | n/a | Magnussons’s Grain Free (1) |
| KD: ‘Intestinal’ | Hill’s I/D (1), Eukanuba Intestinal (1) | n/a |
| KD; single protein | Brit Care Hypoallergenic Rabbit (1), Hill´s D/D salmon (1), MAC’s mono rabbit (1) | VeggieDog Grain Free (1) Happy Dog Africa (1) |
| KD; hydrolyzed | Purina HA Vegetarian (3), Royal Canin HP (2), Dechra Specific Allergen Management plus (3) | Royal Canin HP (4), Royal Canin ultamino (1) |

**SUPPLEMENTARY MATERIAL 3. Stability of percentage of fecal unconjugated primary bile acids over time in fecal samples stored in -80° C. Samples stored for 1.5 – 4 years (median 2.5).**
