# Supplementary material for: Fecal and Clinical Profiles of Dogs With Chronic Enteropathies Treated With Bile Acid Sequestrants for 5–47 Months: A Retrospective Case Series
Source: J Vet Intern Med. 2025 Aug 20;39(5):e70206. doi: 10.1111/jvim.70206 (PMC12365994; doi:10.1111/jvim.70206)
Supplement: Supplementary file 2 — Data S4: Long‐term follow up data of CE dogs treated with BAS. [file JVIM-39-e70206-s001.docx]

**SUPPLEMENTARY MATERIAL 4. Long-term follow up data of CE dogs treated with BAS**

Euthanasia due to NRE or severe flare-up of CE was elected in 4/8 CE-BASR dogs and 4/16 CE+BASR dogs. Four of the CE-BASR dogs are still alive at the time of writing, of which one of the dogs responded to chlorambucil, one to a combination of a home-cooked balanced diet and chlorambucil and one to switching from prednisolone to budesonide. The fourth dog is clinically unstable and under treatment with a hydrolyzed diet, corticosteroids, pregabalin and chlorambucil combined with frequent FMTs administered as a rectal enema.

In dogs responding to BAS, 5/16 dogs are still alive. The 7 dogs euthanized for non-GI disorders had been treated with BAS for 5-47 (median 16) months at the time of death. All dogs could be maintained with 33-67 % lower doses of corticosteroids than before starting BAS. One dog was asymptomatic with cholestyramine treatment until euthanasia 16 months later. The remaining six dogs were markedly more stable, with fewer and milder flare-ups, improved fecal scores compared to baseline and decreased frequency of defecation until time of death. The CE+BASR dogs still alive at the time of writing had a median (range) CIBDAI of 2.5 (2–3) at the last follow-up visit within 1-6 months of writing, which is lower than their corresponding baseline median (range) CIBDAI of 5 (4-8). At that visit, the dogs had been treated with BAS for a median (range) of 18 (6-44) months. Nocturnal defecations had stopped in affected dogs and flare-ups occurred less frequently in all dogs.
